# Calculating the individual probability of successful ocriplasmin treatment in eyes with vitreomacular traction–Validation and refinement of a multivariable prediction model

**DOI:** 10.1371/journal.pone.0270120

**Published:** 2022-07-25

**Authors:** Christoph Paul, Hans-Helge Müller, Thomas Raber, Thomas Bertelmann, on behalf of the EXPORT study group

**Affiliations:** 1 Department of Ophthalmology, Philipps-University Marburg, Marburg, Germany; 2 Institute of Medical Bioinformatics and Biostatistics, Philipps-University Marburg, Marburg, Germany; 3 Oxurion NV, Leuven, Belgium; 4 Thomas Bertelmann M.D., Department of Ophthalmology, University Medical Centre Goettingen, Goettingen, Germany; National Taiwan University Hospital, TAIWAN

## Abstract

**Purpose:**

To evaluate a multivariable model predicting the individual probability of successful intravitreal ocriplasmin (IVO) treatment in eyes with vitreomacular traction (VMT).

**Methods:**

Data from three prospective, multicenter IVO studies (OASIS, ORBIT, and INJECT) were pooled. Patients were included if they were treated for a symptomatic VMT without a full-thickness macular hole. A prediction model for VMT resolution using the factors ‘age’ and ‘horizontal VMT diameter’ was validated by receiver operating characteristic analysis and according to grouped prediction after calibration. Multivariable regression analysis was performed to check robustness and explore further improvements.

**Results:**

Data from 591 eyes was included. In the univariate analysis all key factors (age, gender, VMT diameter, lens status, ERM) significantly correlated to treatment success. The prediction model was robust and clinically applicable to estimate the success rate of IVO treatment (AUC of ROC: 0.70). A refinement of the model was achieved through a calibration process.

**Conclusion:**

The developed multivariable model using ‘horizontal VMT diameter’ and ‘age’ is a valid tool for prediction of VMT resolution upon IVO treatment.

## Introduction

In vitreomacular traction (VMT), an anomalous incomplete posterior vitreous detachment process leads to foveal intraretinal structural changes, which may be accompanied by the development of a full-thickness macular hole (FTMH) or an epiretinal membrane (ERM) [1].

There are currently four main approaches in treating VMT: observation or ‘watchful waiting’ for a possible spontaneous VMT resolution; pars plana vitrectomy; pneumatic vitreolysis involving the intravitreal injection of an expandable gas; and enzymatic vitreolysis involving the intravitreal injection of ocriplasmin (Jetrea®, Oxurion, Leuven, Belgium). These treatment options all aim to release the pathognomonic traction in VMT [2] and have distinct advantages and disadvantages for the treatment of VMT. Observation has its value, as a spontaneous resolution of VMT is observed in 20–40% of cases [3, 4], depending on the observation duration. However, this process may take up to two years [3] and VMT is accompanied by visual impairment and vision-related reduced quality of life [5]. Vitrectomy offers a success rate of virtually 100%, yet is the most invasive and most technically demanding option. The intravitreal injection of gas or ocriplasmin is simple to perform, but in metanalyses, their success rate has been limited to 50–80% (gas) [6, 7] and 30–40% (ocriplasmin) [8] within the first 28 days after injection. While enzymatic vitreolysis is a relatively safe procedure [8, 9] however with side effects like ellipsoid zone changes, subretinal fluid and retinale tears [10], the first prospective studies on pneumatic vitreolysis have been terminated early due to a high rate of rhegmatogenous retinal detachments [7].

When judging these options by their success rates, one has to consider that the success rates all of these options–except for vitrectomy–are dependent on patient characteristics. The most comprehensive data on this have been generated for IVO, for which randomized controlled trials have been performed, in contrast to intravitreal gas injection or observation. Younger age, female gender, smaller adhesion diameter (as a surrogate for adhesion area [11]), phakic lens status, and the absence of an ERM have been linked to a higher frequency of VMT resolution after IVO treatment. Of these factors, lens status is highly correlated to age and is possibly not an independent predictor [12]. Additionally, specific angles between vitreous and retina show a correlation to IVO treatment success [13]. However, they are more demanding to measure and weaker in their predictive value.

The predictive factors are potentially overlapping for IVO, spontaneous resolution and pneumatic vitreolysis. Younger age (in adult patients) has been described as a positive predictor for all three options [3, 8, 14]. The lack of an ERM and female gender are positive predictors for both IVO and pneumatic vitreolysis [8, 14]. Foveal vitreous adhesion angles have been linked to vitreous detachment both after IVO and spontaneous resolution [13, 15].

Knowledge of the predictive factors is therefore important in the clinical decision-making process. Judging the individual probability of successful treatment is complicated by the correlation of the factors (e.g. age and lens status). To approach this problem in the case of enzymatic vitreolysis, we previously used the dataset of an observational study on IVO, the EXPORT study [16], to generate a multivariable predictive model [12]. We determined that age and VMT diameter were the strongest predictors of treatment success and their combination allowed the calculation of an individual probability of success. While this model showed promising results in the performed cross-validation, the external validity could not be assessed due to a missing independent patient cohort [12].

We were subsequently granted access to the anonymized data of three separate prospective, multicenter studies on IVO: the OASIS [17], ORBIT [18], and INJECT [19] studies. We used the data from this independent cohort in our current study to assess the validity and to explore a refinement of our previously-described prediction model.

## Methods

We performed a pooled analysis of patients treated with IVO for VMT in multicenter prospective clinical studies to validate a multivariable predictive model for IVO success. This analysis was approved by the institutional review board (“Ethikkommission der Universitätsmedizin Göttingen” No. 28/1/19, dated 31/Jan/2019). Patients were included from three separate studies:

- ‘Ocriplasmin for Treatment for Symptomatic Vitreomacular Adhesion Including Macular Hole’ (OASIS) [17], a Phase 3b, randomized, sham-controlled, double-masked, multicenter clinical trial.
- ‘Ocriplasmin Research to Better Inform Treatment’ (ORBIT) [18], a Phase 4 multicenter, prospective, observational study.
- ‘Investigation of JETREA in Patients Confirmed Vitreomacular Traction’ (INJECT) [19], a Phase 4, multicenter, prospective, observational study.

Written informed consent was obtained from all patients for trial participation as well as subsequent secondary analyses of the data prior to study start. All methods were carried out in accordance with local, national and international guidelines and regulations. Patients in these studies were diagnosed with a VMT and received IVO. All three studies included patients with pure VMT and VMT associated with an FTMH. The aim of our analysis was the evaluation of a multivariable model to predict the probability of VMT resolution upon IVO treatment in eyes without an FTMH. Therefore, patients were included in our analysis if they fulfilled the following criteria:

- Diagnosed with a symptomatic VMT and had received IVO
- Treatment success had been assessed 28 ± 5 days after injection (to assess therapeutic success defined as a complete cleavage of the posterior vitreous cortex from the internal limiting membrane in the scanned OCT frame–in accordance with the MIVI trials [2])
- The key variables (*age*, *gender*, *horizontal diameter of vitreomacular attachment*, *presence of an ERM*, *presence of an FMTH)* available
- Absence of FTMH

A total of 920 eyes with symptomatic VMT were treated with IVO in the OASIS (145), INJECT (295) and ORBIT (480) studies with known outcome. The data of these treated patients were provided in plain numbers with a performed grading for the intraocular parameters (VMT-diameter, presence of ERM, presence of FTMH, lens status). The original OCT scans were not available. This dataset was screened for patients matching the inclusion criteria. The VMT-diameter was not provided for 113 patients (OASIS: 11, INJECT: 83, ORBIT: 19). Therefore, these patients missing the key factor of the multivariable model were excluded. A further 216 patients were excluded because they were treated for a VMT with associated FTMH, for which the multivariable model was not developed. Thus, 591 patients remained for statistical analysis. Specific angles of the vitreoretinale interface [13] were not analyzed because they were not assessed in the founding studies, and OCT scans were not available to ex post perform these measurements

The focus of the statistical analysis was the evaluation of a previously described [12] multivariable model. The analysis was conducted in R 3.4.0 (R core team, 2017) [20]. First, univariate regression analysis was conducted to verify the association of the previously-described predictors. Next, to judge the diagnostic ability of the model, the *‘pROC’ package (v1.16.2) [21] was used to perform a receiver operating characteristic (ROC) curve analysis. For this, the individual probability of successful treatment predicted by the* previously developed model [12] (‘original EXPORT model’) was combined with the known outcome of success of each patient (of the ORBIT, OASIS and INJECT pool) to plot the specificity (with inverted axis) versus the sensitivity, with the threshold being the predicted probability. Then, the previously developed model was calibrated), thus the transferability of the model to a new patient cohort, was performed using the R package ‘givitiR’ (v1.3) [22]. In this package, the calculated predictions of treatment success (with the ‘original EXPORT model’) were related to the true probabilities of success (of the ORBIT, OASIS and INJECT pool) in a second logistic regression model, based on a polynomial transformation of the predictions. This linear calibration results in a constant β_0_ and a regression coefficient β_1_ which were added to the model. This ‘calibrated model’ remains the same relation of the factors age and VMT diameter as the ‘original EXPORT model’, since the same β_1_ coefficient is used for both factors. A ‘calibration belt’ is plotted as a graphical interpretation of these calculations, conveying the uncertainty in the estimated relationship between predictions and the probabilities of success. Of note, the EXPORT model uses the natural logarithm (ln) of the horizontal VMT diameter. Hence, the ln(VMT diameter) is presented for the multivariable analyses (e.g. Table 2). In addition to this external validation, a new multivariable logistic regression analysis was performed on the patient dataset for further freedom in model generation. Variables were added if there was a significant additional effect for discrimination based on a level of the p value and the ‘Akaike information criterion’ (AIC). To further evaluate the multivariable models, the individual probability of success was calculated for every patient. For this, odds were computed with the parameters given in Table 2 using Eq (1):

$${Odds}_{IVO-Success}=e^{Intercept}*{OR}_{age}^{years}*{OR}_{ln\;diameter}^{\ln\left(\mu m\right)}$$ (1)

Odds were then converted to probabilities using Eq (2):

$${Probability}_{IVO-Success}=\frac{{Odds}_{IVO-Success}}{\left({Odds}_{IVO-Success}+1\right)}$$ (2)

Patients were grouped by their predicted success rate and mean observed success rates were calculated for these groups.

## Results

In our study data of 591 patients was analyzed. Of these 224 were male (37.9%) and 367 (62.1%) female. The mean age (± SD) was 73.1 ± 8.5 years (range 38–94). Of the included eyes, 382 (64.6%) were phakic and 197 (33.3%) were pseudophakic. Lens status was not documented for 12 (2.0%) eyes. The mean horizontal VMT diameter was 482 ± 391 (range 40–3210) μm. An ERM was present in 127 (21.5%) eyes. Out of 591 eyes, 227 (38.4%) were treated successfully with IVO. A detailed description of the parameters in the subgroups of successfully and unsuccessfully treated eyes is given in Table 1.

**Table 1:** Univariate regression analysis results of the analysed variables.

Variable	VMT resolution	VMT persistence	OR (95% CI)	p value
	n = 227	n = 364		
**Age [years]**	69.6 ± 8.6	75.3 ± 7.7	0.917 (0.896 to 0.938)	< 0.0001
**VMT diameter [μm]**	411 ± 340	526 ± 414	0.9990 (0.9984 to 0.9996)	< 0.0001
**Gender [male]**	67 (29.5%)	157 (43.1%)	0.552 (0.387 to 0.783)	< 0.0001
**Phakic**	169 (74.4%)	213 (58.5%)	1.949 (1.354 to 2.830)	< 0.0001
**ERM**	21 (11.5%)	101 (27.7%)	0.337 (0.207 to 0.531)	< 0.0001

VMT (vitreomacular traction) resolution = treatment success; VMT persistence = treatment failure.

Quantitative parameters (age, VMT diameter): mean ± SD.

Binary parameters (gender, lens status, ERM formation): number (percentage).

The corresponding odds ratio (OR) with a 95% confidence interval (CI) per one-unit difference for quantitative/continuous parameter, respectively, presence versus absence of the listed results for the binary parameters (e.g., male versus female) and the P-value (P) for the difference in odds for/of VMT resolution are shown.

The univariate regression analysis revealed the all observed parameters (age, VMT diameter, gender, lens status, ERM) correlated significantly to treatment success (Table 1). A positive correlation was found for phakic lens status and a negative correlation was found for age, VMT diameter, presence of an ERM, and male gender.

**Fig 1:**
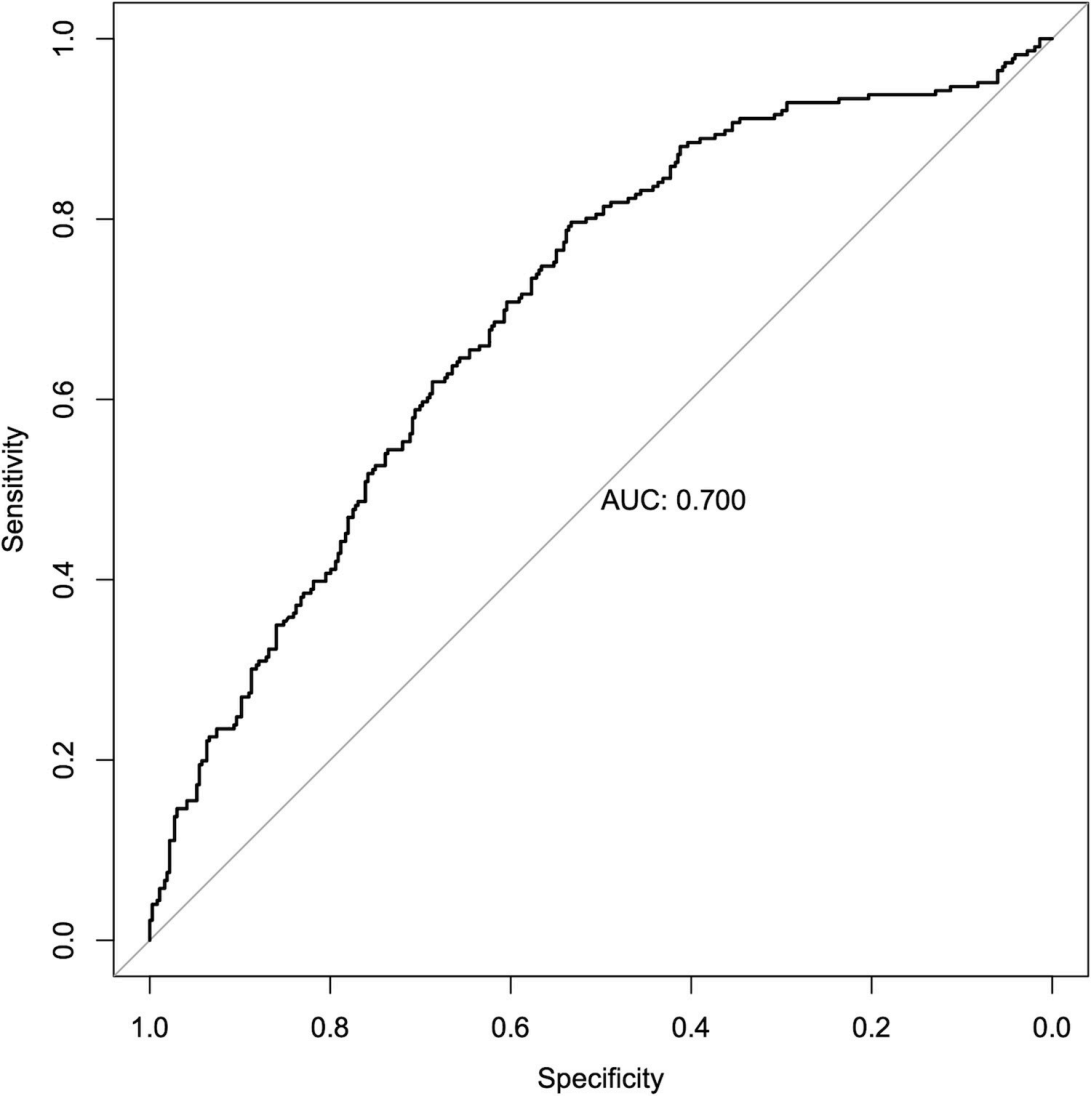
Receiver operating characteristic (ROC) curve of the prediction models. The black line depicts the ROC curve both for the ‘EXPORT’ as well as its monotonously transformed ‘calibrated EXPORT’ model. The sensitivity (y-axis) is plotted as a function of the specificity (x-axis). The area under the curve (AUC) is 0.700.

To evaluate the previously published multivariable model [12], the individual probability of successful IVO treatment was calculated with this model. The resulting ROC curve with the specificity (with inverted axis) versus the sensitivity is given in Fig 1. The corresponding area under this ROC curve (AUC) was 0.70.

**Fig 2:**
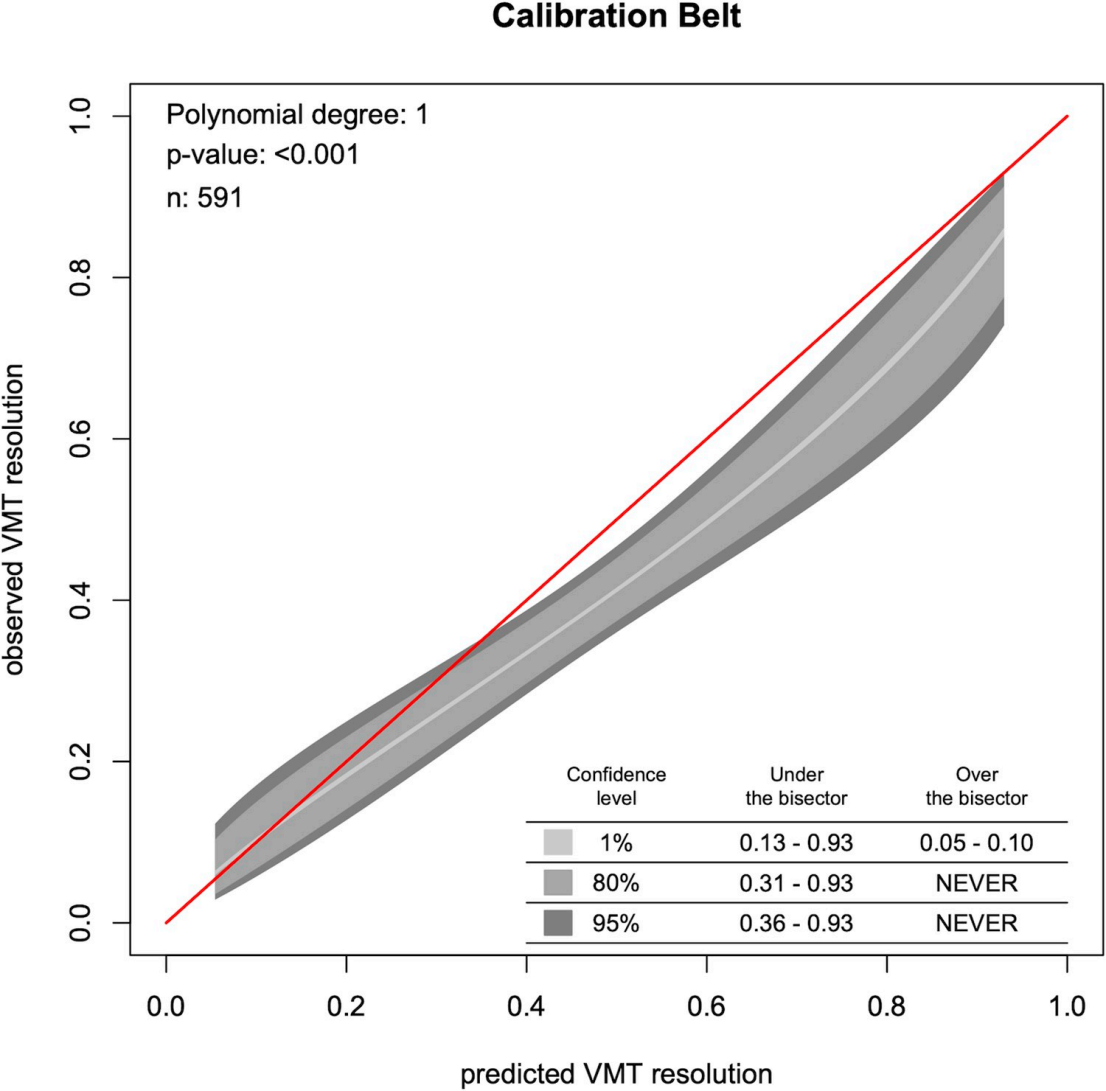
Calibration belt of the predicted VMT resolution by the EXPORT model and the observed VMT resolution in the current pooled patient cohort. The red line depicts a theoretical perfect correlation. The calibration belt is plotted in grey, consisting of the 95% (dark grey), 80% (grey) and 1% (light grey) confidence interval. It conveys the uncertainty in the estimated relationship between predictions and the probabilities of the true response, and was calculated using the ‘givitiR’ R package. This package utilizes a logistic regression model, based on a polynomial transformation (a linear transformation in this application), which relates the prediction of the EXPORT model to the true probabilities observed.

In the next step, the calibration was investigated. The calibration of the model was relatively good (p < 0.001 for testing differences from β_1_ = 1). The resulting ‘calibration belt’ is given in Fig 2. The calculated factors β_0_ (-0.352) and β_1_ (0.827) were used to calibrate and consequently improve the model. The parameters of the resulting ‘calibrated model’ are displayed in Table 2.

**Table 2:** Comparison of the multivariate logistic regression analyses for successful vitreomacular traction resolution.

Variable	Original EXPORT model	Calibrated EXPORT model OR	Recalculated model
			OR, (CI); p value
	OR, (CI); p value		
**Intercept**	10.5133	8.3448	8.4967
**ln(diameter [μm])**	0.38331, (0.218 to 0.674); 0.0009	0.45236	0.6169, (0.467 to 0.810); <0.0001
**Age [years]**	0.93379, (0.895 to 0.974); 0.0013	0.94491	0.91924, (0.898 to 0.940); <0.0001

Original EXPORT model: multivariate model generated from the EXPORT study.

Calibrated EXPORT model: calibrated model derived from the original EXPORT model.

Recalculated model: model based on calculations of the combined INJECT/OASIS/ORBIT studies.

Odds ratios (OR), 95% confidence interval (CI) and p values of the difference in odds of VMT resolution.

To judge the robustness of the model, a multivariable model generation was performed using the pool of 591 patients. The parameters of the newly formed model (‘recalculated model’) are described in Table 2, as a comparison to the original model from the EXPORT study. Of interest, there was a minimal increase in the influence of age (OR age: 0.92 vs 0.93), yet some decrease for the VMA-diameter (OR ln(VMA-diameter): 0.61 vs 0.38).

**Fig 3:**
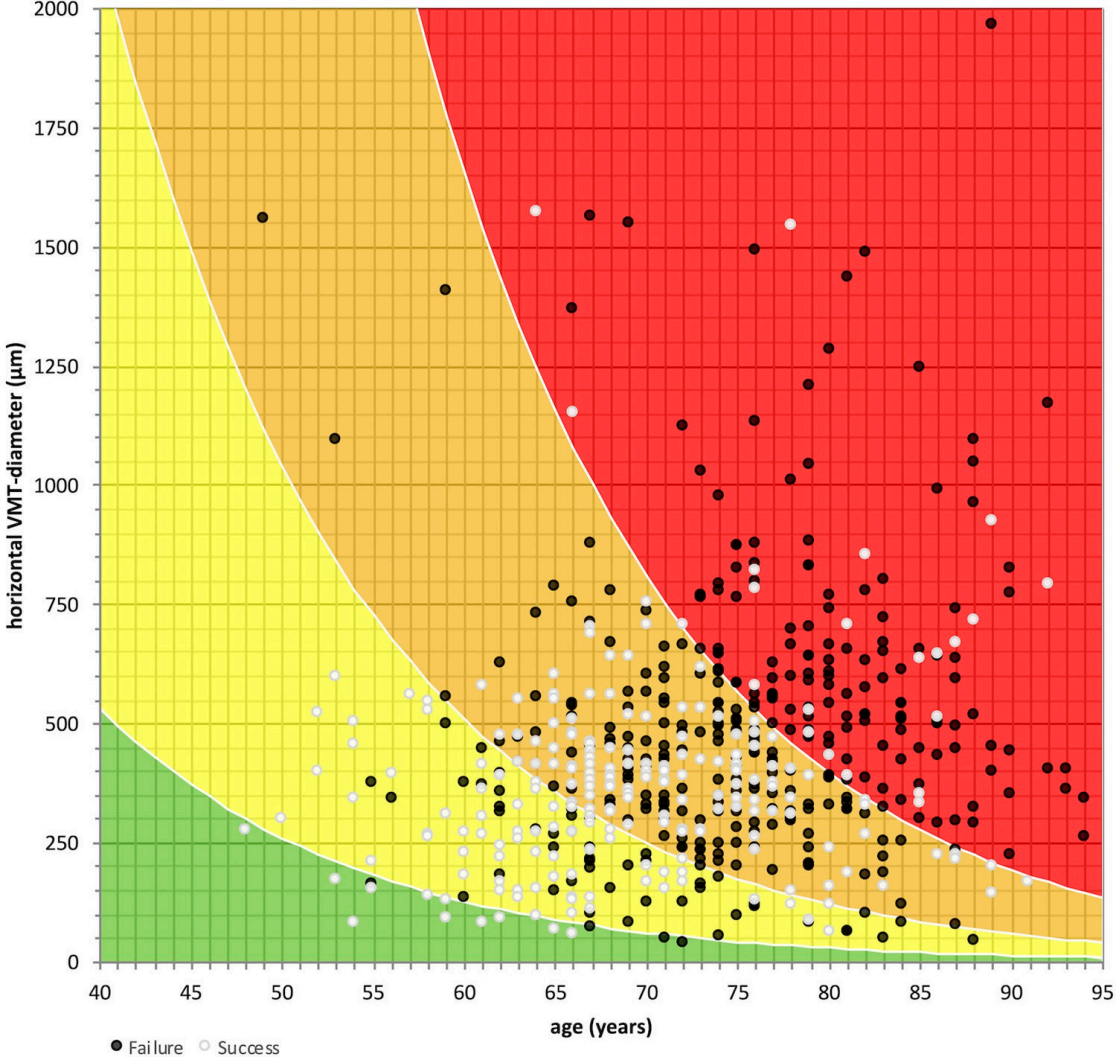
Two-dimensional plot of the probability of successful treatment with ocriplasmin. The calibrated EXPORT multivariable model was used to calculate the probability of successful treatment (grouped in 25% intervals; red: intravitreal ocriplasmin (IVO) success group: 0% to 25%, orange: 25% to 50%, yellow: 50% to 75%, and green: 75% to 100%) with a dependence on age (x-axis) and horizontal vitreomacular traction (VMT) diameter (y-axis). Overlaid are all 591 patients arranged by their age (x-axis) and horizontal VMT diameter (y-axis). The treatment outcome is displayed by the marking: patients in which IVO treatment was successful (VMT resolution) are marked by white dots, those in which treatment was not successful (VMT persistence) are marked with black dots.

**Fig 4:**
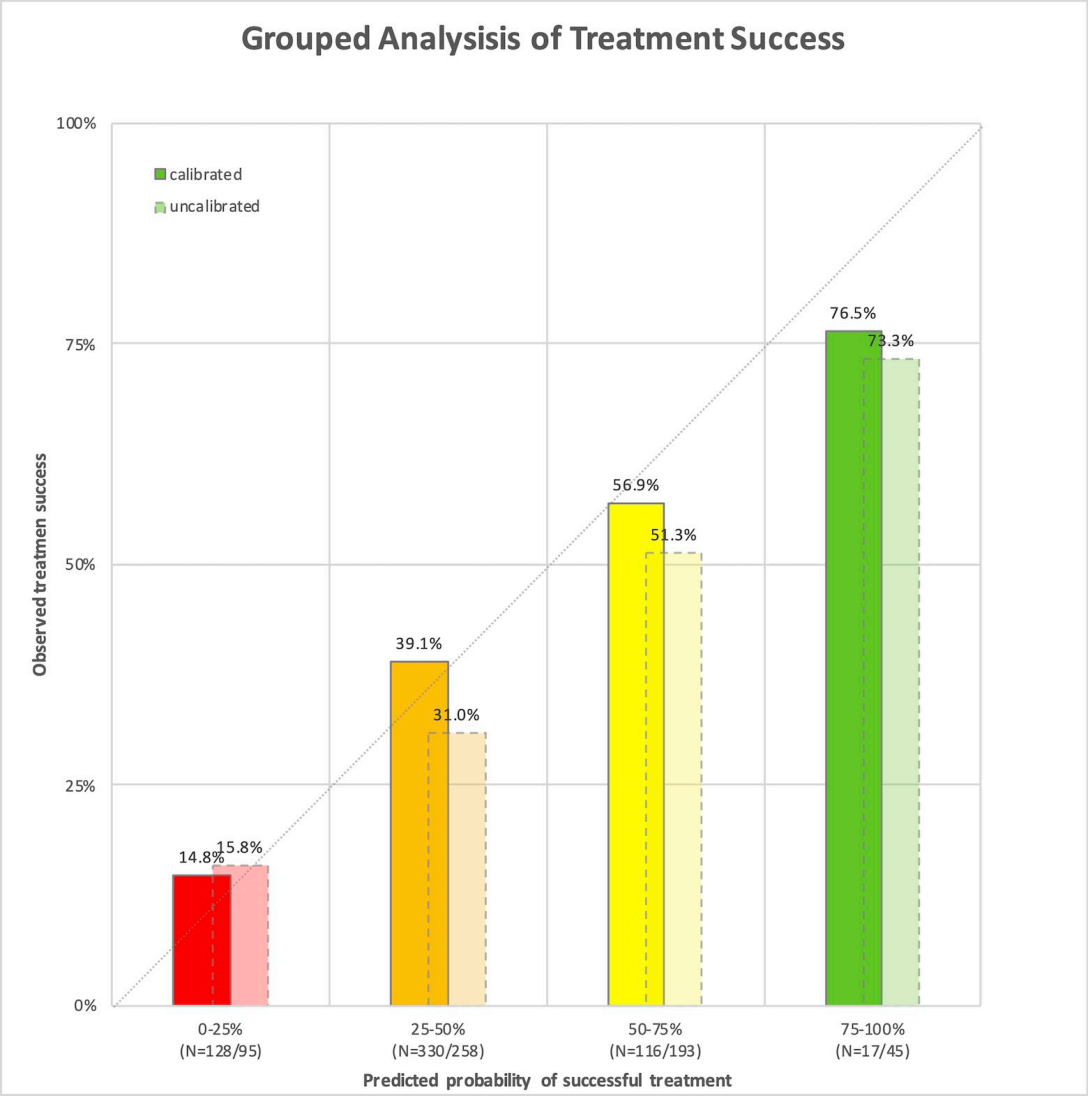
Correlation of the observed success rate to the predicted probability of success from the multivariable prediction models (original, calibrated and recalculated). The individual probability of ocriplasmin treatment success was calculated for every patient. Patients were grouped by their calculated success probability (grouped in 25% intervals; red: intravitreal ocriplasmin (IVO) success group: 0% to 25%, orange: 25% to 50%, yellow: 50% to 75%, and green: 75% to 100%). The calculation was performed with the calibrated model (dark colors, continuous border) and uncalibrated EXPORT (light colors, broken border) models. Number (n) of patients per interval in both models is given at the x-axis.

To envision the developed multivariable model, patients were grouped by their predicted probability of IVO success in 25% intervals. The assignment to these groups, dependent on the age and horizontal diameter of each patient, were computed with Eq (1) and Eq (2) with the respective parameters (Table 2). For the calibrated EXPORT model this assignment is visualized in Fig 3. In each group, the mean observed success rate was calculated (Fig 4). It was within the calculated range for both models–with both variants a prediction of treatment success was possible. Of note, the ROC curve for both the original EXPORT model and the calibrated EXPORT is the same, as the ROC analysis is not affected by the monotonous transformation in the calibration process.

## Discussion

We had previously conducted a multivariable analysis of prognostic factors in IVO and demonstrated that the combination of patient age and horizontal VMT diameter was the strongest predictor of treatment success [12]. We had used this information to create a two-factor predictive model, which we were able to validate in this current study.

Due to the large number of patients (591) in the combined OASIS, INJECT and ORBIT cohort, we were able to analyze a very diverse population of eyes with focal and broad VMT. The observed success rate (38.4%) was lower than in the included studies (OASIS: 41.7% [17], INJECT: 40.7% [19], ORBIT: 45.8% [18]), due to the exclusion of eyes with FTMH–an known positive predictor of VMT resolution in enzymatic vitreolysis [8].

In comparison to the previous EXPORT study [12] (47.3%), the here observed success rate was lower. This difference might be ascribed to different patient characteristics in the analyzed populations: Patients in the EXPORT study were slightly younger (72.7 ± 8.9 vs 73.1 ± 8.5 years, respectively), had a higher frequency of women (70.7% vs 62.1%), and smaller VMT-diameter (467 ± 418 μm vs 482 ± 391 μm)–all known positive predictors of VMT resolution. The success rate was, however, higher than the initial ‘Microplasmin for Intravitreous Injection’ (MIVI) studies [2] (26.5%) and within the range of published observational studies (range: 0–71%) [8].

The first objective of our study was to determine the validity of the predictive model. For this, an ROC analysis was performed. In this independent cohort, the model performed comparably to the cross-validation performed in the previous analysis, with an AUC of 0.700 versus 0.736 in previously-done leave-one-out cross-validation [12]). Hence, external validity has been demonstrated for this model.

The second objective of our study was to improve the model using the larger patient cohort. The calibration processes led only to minor changes in the model and the model was capable of predicting the success rate of patients with VMT. To evaluate the robustness of the model, we again performed a model generation in the combined patient pool of OASIS, ORIBT, and INJECT datasets. The resulting model (the ‘recalculated model’) was similar to the original EXPORT model. In the recalculated model, VMT diameter had a slightly less negative impact on treatment success than in the original model (OR of the ln(VMT diameter): 0.62 vs 0.38) and age a slightly stronger negative effect (OR: 0.919 vs 0.934), however, both values of the recalculated model were within in the confidence interval of the original EXPORT prediction model. In the clinical applicability, these changes had only a minor influence with an AUC change in the ROC analysis of the recalculated model of 0.707 versus 0.700 in the original model and a similar prediction in the grouped analysis. Of note, with this larger patient cohort, a three-factor prediction model of age, VMT diameter and presence of ERM, as well as a four-factor prediction model of age, VMT diameter, presence of ERM, and gender were significant. Implementation of these additional factors did however not have a major impact on the clinical applicability (AUC three factor model: 0.718, AUC four factor model: 0.720). Also, decrease of the ‘Akaike information criterion’ was observed with the implementation of factor 3 (AIC = 699) and 4 (AIC = 697) suggesting no benefit of these models in comparison to the 2-factor model (AIC = 716).

Of interest, the factor of lens status was not significant in the multivariate analysis in contrast to the univariate analysis. This supports the hypothesis that it is not separate predictor in this context but a surrogate of patient age [12], which it is correlated to.

As indicated in the grouped analysis, a distinction of patients with very high (>75%) and very low (<25%) success rate was possible with all variants of the prediction model. Since the external validation of the model was performed successfully for the EXPORT model we advise the use of the calibrated EXPORT model (Table 2).

Our study has three main limitations. Firstly, our predictions were only done in patients without FTMH. Therefore, no conclusion may be drawn for those patients with VMT and associated macular hole. A recent study investigated the use of the original EXPORT model in patients with FTMH [23]: the model predicted the probability of VMT-resolution but not the probability of macular hole closure, the relevant endpoint for these patients. Sole predictor of macular-hole closure was a macular hole diameter of <250 μm, which was positively correlated to treatment success. Secondly, possible negative impacts of ocriplasmin treatment are not implemented into the prediction model. Even though IVO has been described as a relatively save procedure [8, 9], complications may include retinal tears, retinale detachment, progression of VMT to macular hole, lentodonesis, dyschromatopsia, subretinal fluid, OCT changes in the ellipsoid zone, and changes in the electroretinography [10, 24]. Thirdly, the distribution of individual success rates found in this cohort resembles a normal distribution with a median predicted VMT resolution of 36.4%. In the analyzed cohort only 145 of 591 patients belonged to the groups with either very low (<25%, N = 128) or very high (>75%, N = 17) probability of success. More than half of all patients had a probability of success in the second quartile of 25 to 50%. Consequently, the prediction model will in clinical practice often return probabilities within this range, which may be less conducive for decision making than very high or very low probabilities.

The strength of this model is that it allows the robust estimation of treatment success in IVO. The establishment of this model was founded on a very large and consistent patient cohort for its indication, and the model performed well in the validation. Even though they are limited in number, patient sub-groups with higher-than-previously-described success rates exist and may be identified using the two-factor model described here. This is of special clinical interest as ocriplasmin is nowadays less commonly used due to its limited success rates [25], but these subgroups might benefit from an ocriplasmin treatment.

Further research is warranted to predict the probability of spontaneous resolution of VMT and the probability of successful pneumatic vitreolysis. The known overlapping predictive factors with IVO support the idea that similar models might also be achievable for these treatment options.
